# Supplementary material for: MLL-fusion-driven leukemia requires SETD2 to safeguard genomic integrity
Source: Nat Commun. 2018 May 18;9:1983. doi: 10.1038/s41467-018-04329-y (PMC5959866; doi:10.1038/s41467-018-04329-y)
Supplement: Supplementary file 1 — Supplementary Information [file 41467_2018_4329_MOESM1_ESM.pdf]

Supplementary Information to the publication

# MLL-fusion-driven leukemia requires SETD2 to safeguard genomic integrity

Anna Skucha[1,2], Jessica Ebner[2], Johannes Schmöllerl[2], Mareike Roth[3], Thomas Eder[2], Adrián César-Razquin[1], Alexey Stukalov[1], Sarah Vittori[1], Matthias Muhar[3], Bin Lu[4], Martin Aichinger[3], Julian Jude[3], André C. Müller[1], Balázs Győrffy[5], Christopher R. Vakoc[4], Peter Valent[6], Keiryn L. Bennett[1], Johannes Zuber[3*], Giulio Superti-Furga[1,7*], Florian Grebien[2,8*#]

[1] CeMM Research Center for Molecular Medicine of the Austrian Academy of Sciences, Vienna, Austria

[2] Ludwig Boltzmann Institute for Cancer Research, Vienna, Austria

[3] Research Institute of Molecular Pathology, Vienna, Austria

[4] Cold Spring Harbor Larboratory, Cold Spring Harbor, USA

[5] MTA TTK Lendület Cancer Biomarker Research Group, Institute of Enzymology, Second Department of Pediatrics, Semmelweis University, Budapest, Hungary

[6] Department of Internal Medicine I. Division of Hematology & Hemostaseology, Ludwig Boltzmann Cluster Oncology, Medical University of Vienna; Austria

[7] Center for Physiology and Pharmacology, Medical University of Vienna, Austria

[8] Institute for Medical Biochemistry, University of Veterinary Medicine, Vienna, Austria

* these authors contributed equally to this work

[#] lead contact: Florian Grebien:

**florian.grebien@lbicr.lbg.ac.at**, **florian.grebien@vetmeduni.ac.at**

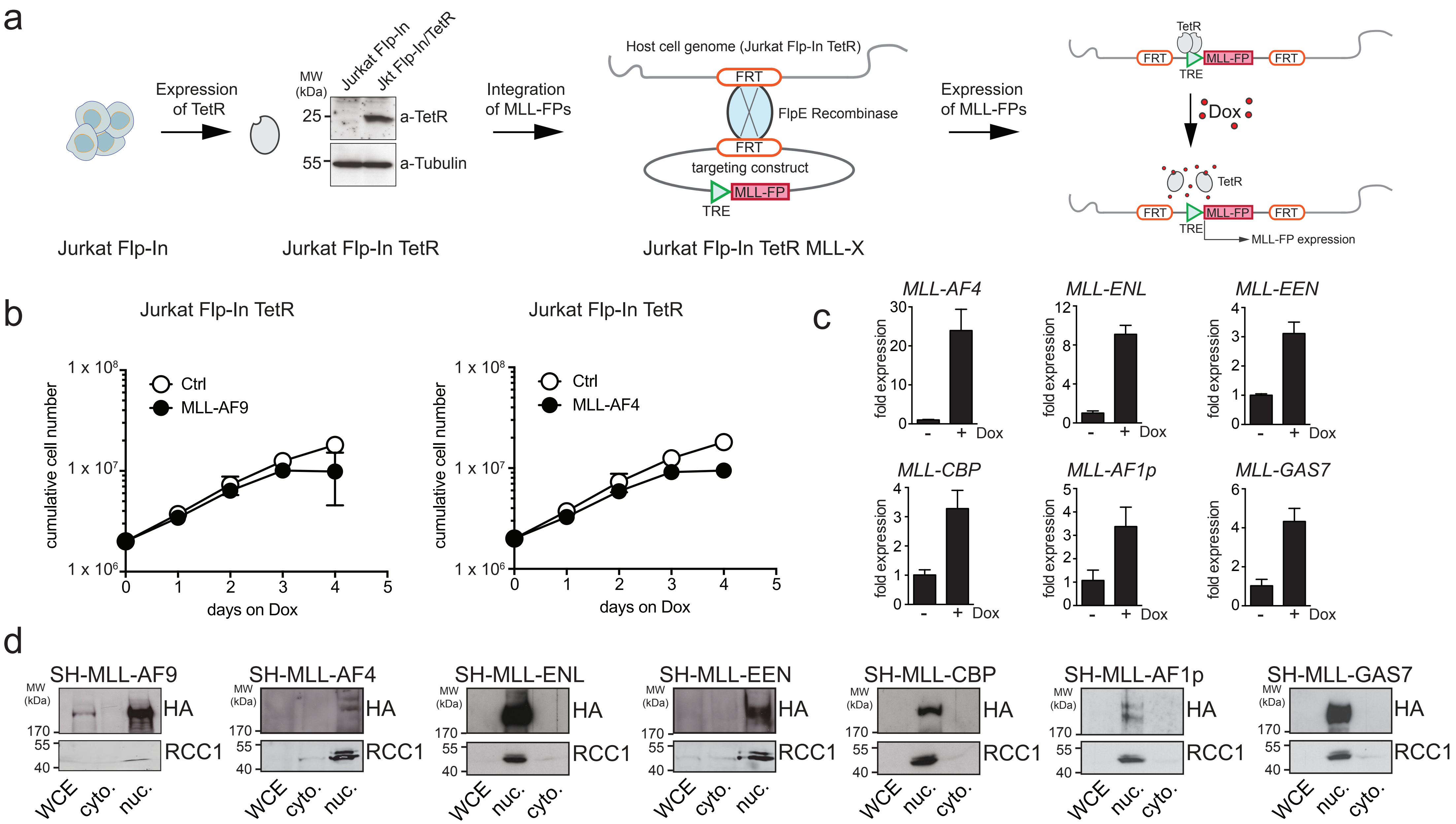


**Supplementary Figure 1. Generation and characterization of isogenic cell lines for expression of affinity-tagged variants of MLL-fusion proteins. (a)** Schematic illustration of the workflow for establishing the cell line models for affinity purification of MLL fusion proteins. The Flp-In system involves introduction of a Flp Recombination Target (FRT) site into the genome of the Jurkat cell line. An expression vector encoding for MLL-fusion protein is integrated into the genome via Flp recombinase-mediated DNA recombination at the FRT site. **(b)** Growth curves of Jurkat Flp-In cell lines expressing indicated MLL-fusion proteins (mean±s.d. n=3). **(c)** Cells expressing Strep-HA (SH)-tagged MLL fusion proteins were treated with Dox for 24h and transgene expression was monitored by qPCR (mean±s.d. n=3). **(d)** Extracts from Jurkat cells expressing indicated MLL-fusion proteins were fractionated and analyzed for expression of hemagglutinin (HA). RCC-1 was used as loading control (WCE: whole cell extract; nuc: nuclear fraction; cyto: cytosolic fraction).

SKUCHA_Supplementary Figure 1

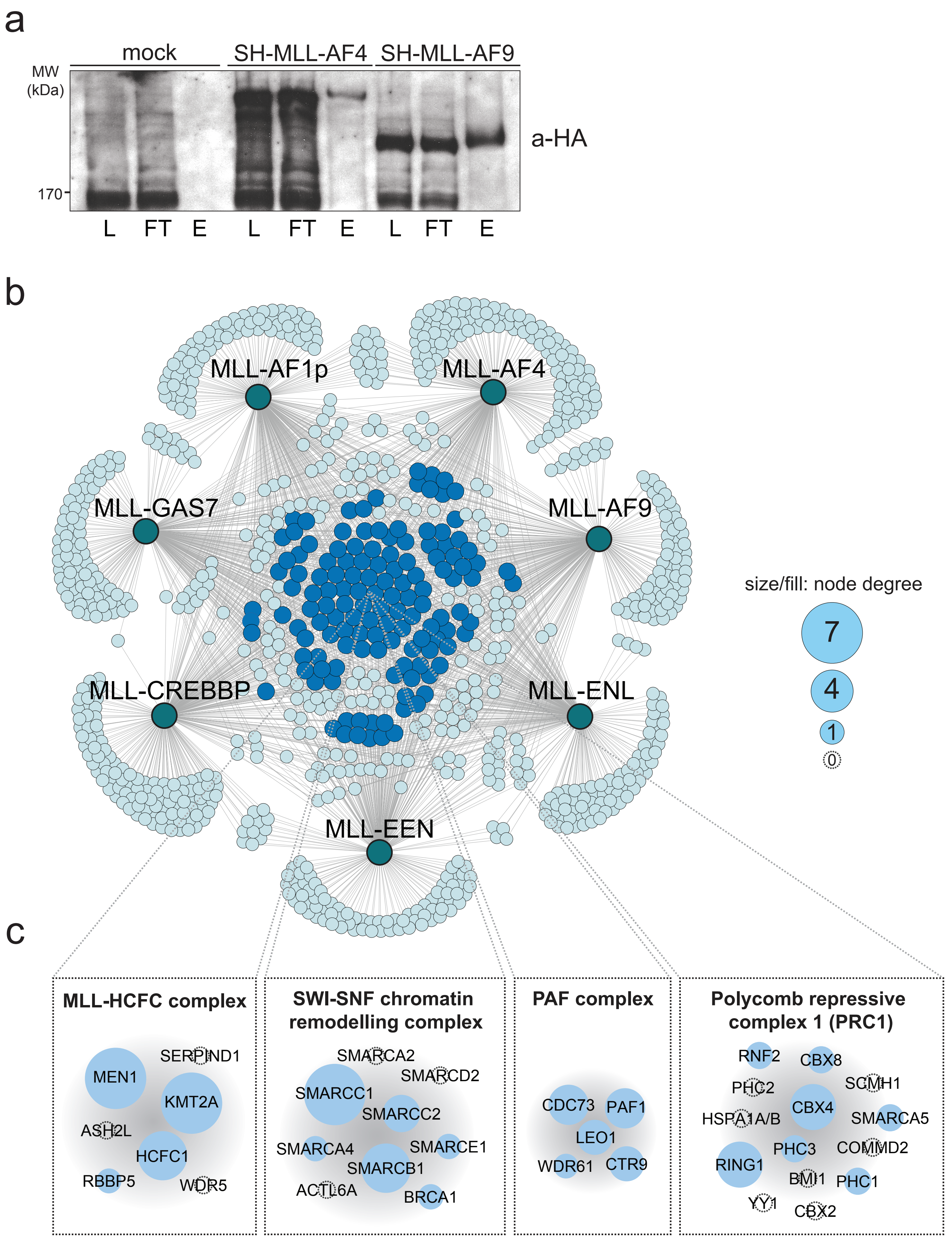


**Supplementary Figure 2. Identification of protein complexes among the MLL-fusion protein-interactome. (a)** Western blot (WB) analysis of lysate (L), flow-through (FT) and eluate (E) from StrepTactin-purifications of indicated affinity-tagged MLL-fusion proteins. **(b)** Network representation of the AP-MS-derived protein-protein interaction network of seven MLL-fusion proteins (dark green nodes), showing the top 300 interactors per bait ranked by p-value (light blue nodes). Nodes labelled in dark blue represent 128 proteins that interact with ≥5 of the 7 MLL-fusion proteins. **(c)** Representation of enriched protein complexes previously reported to be associated with MLL. The size and the color of the nodes correspond to the number of interaction partners.

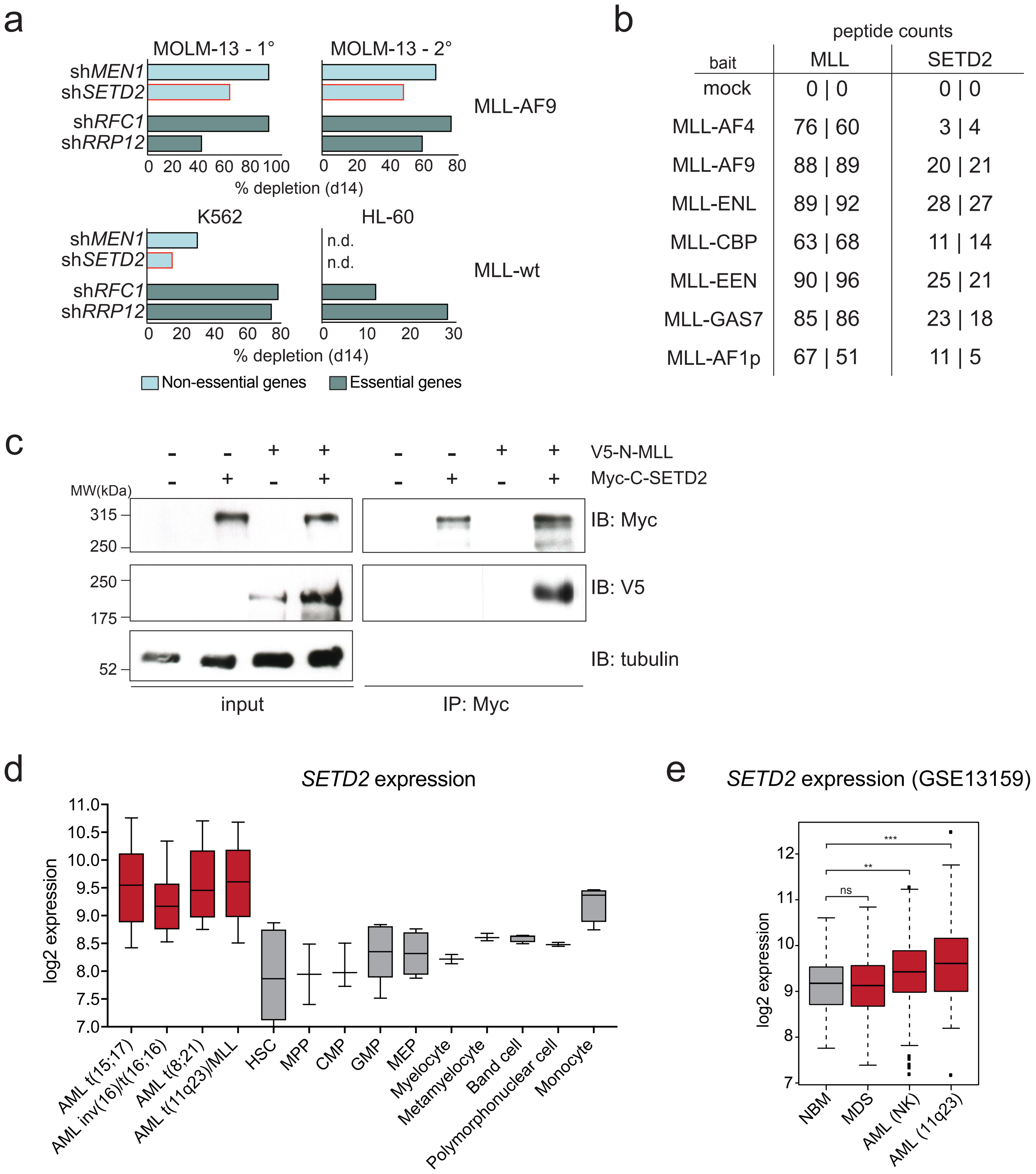


**Supplementary Figure 3. shRNA screening of conserved MLL-interactors identifies SETD2 as a critical effector of MLL-fusion proteins. (a)** Examples of depletion phenotypes of four selected genes in MLL-rearranged vs. MLL-wild type leukemia cell lines. Gene essentiality was assigned based on published datasets. **(b)** Peptide counts for MLL and SETD2 from AP-MS experiments of MLL-fusion proteins. **(c)** HEK293 rtTA3 cells were transiently transfected with indicated constructs. Following immunoprecipitation using anti-Myc beads, eluates (IP) and whole cell extracts (input) were analyzed by immunoblotting with the indicated antibodies. **(d)** Expression levels of SETD2 in leukemia and normal hematopoiesis (bloodspot.binf.ku.dk). **(e)** Expression levels of SETD2 in different AML subtypes (NBM: Normal Bone marrow; MDS: Myelodysplastic Syndrome; AML (NK): Normal Karyotype; AML (11q23): AML harboring rearrangements of the MLL gene). ns, not significant, ** p<0.01, *** p<0.001 (t-test).

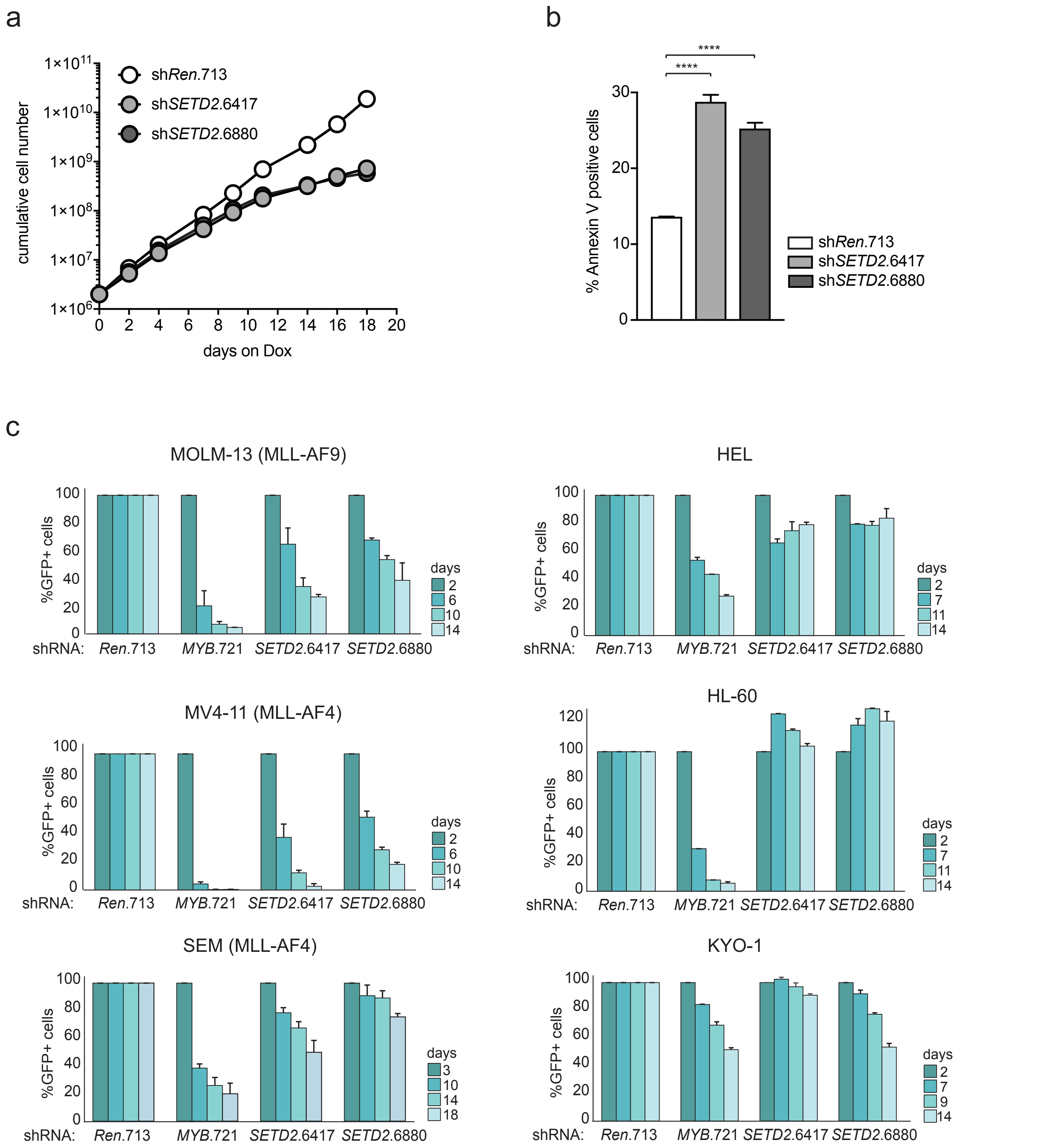


**Supplementary Figure 4. shRNA-mediated knockdown of SETD2 impairs proliferation of human MLL-rearranged cell lines. (a)** Growth curves of MV4-11 cells expressing indicated shRNAs (mean±s.d. n=3). **(b)** Quantification of flow cytometric analysis of apoptosis as measured by Annexin V-staining in MV4-11 cells. Cells were treated with Dox for 11 days and stained according to manufacturer's protocol. **** p<0.0001 (t-test) (mean±s.d. n=3). **(c)** Results of FACS-based competitive proliferation assay shown as the percentage of GFP-positive cells expressing individual *SETD2*-targeting shRNAs in the presence of Dox over 14-18 days in indicated human cell lines (mean±s.d. n=2).

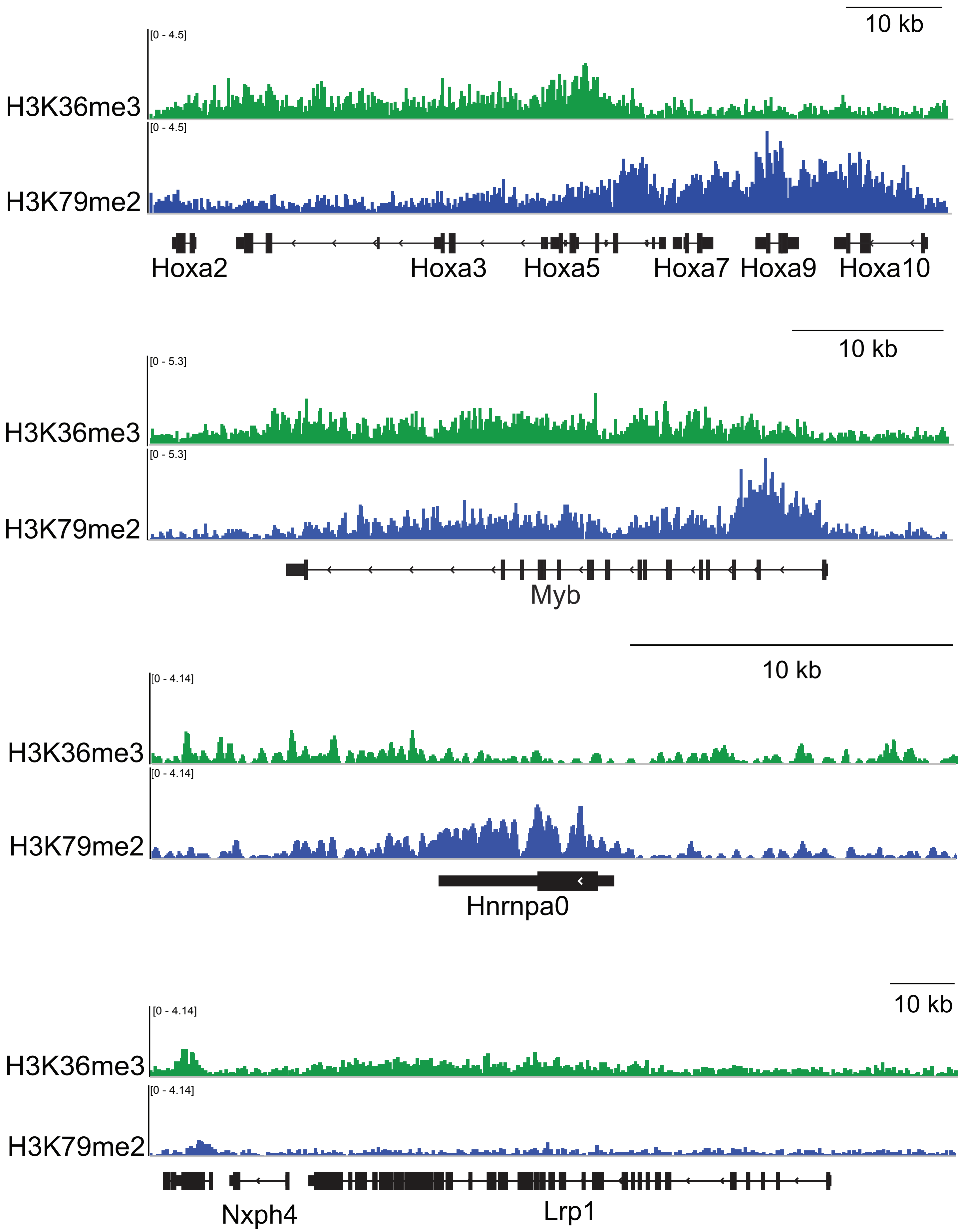


**Supplementary Figure 5. MLL-target genes show high levels of H3K36me3 and H3K79me2.** H3K36me3 (green) and H3K79me2 profiles (blue) of selected MLL-AF9 target genes Hoxa-cluster genes and Myb vs. non-MLL target genes (Hnrnpa0 and Lrp1).

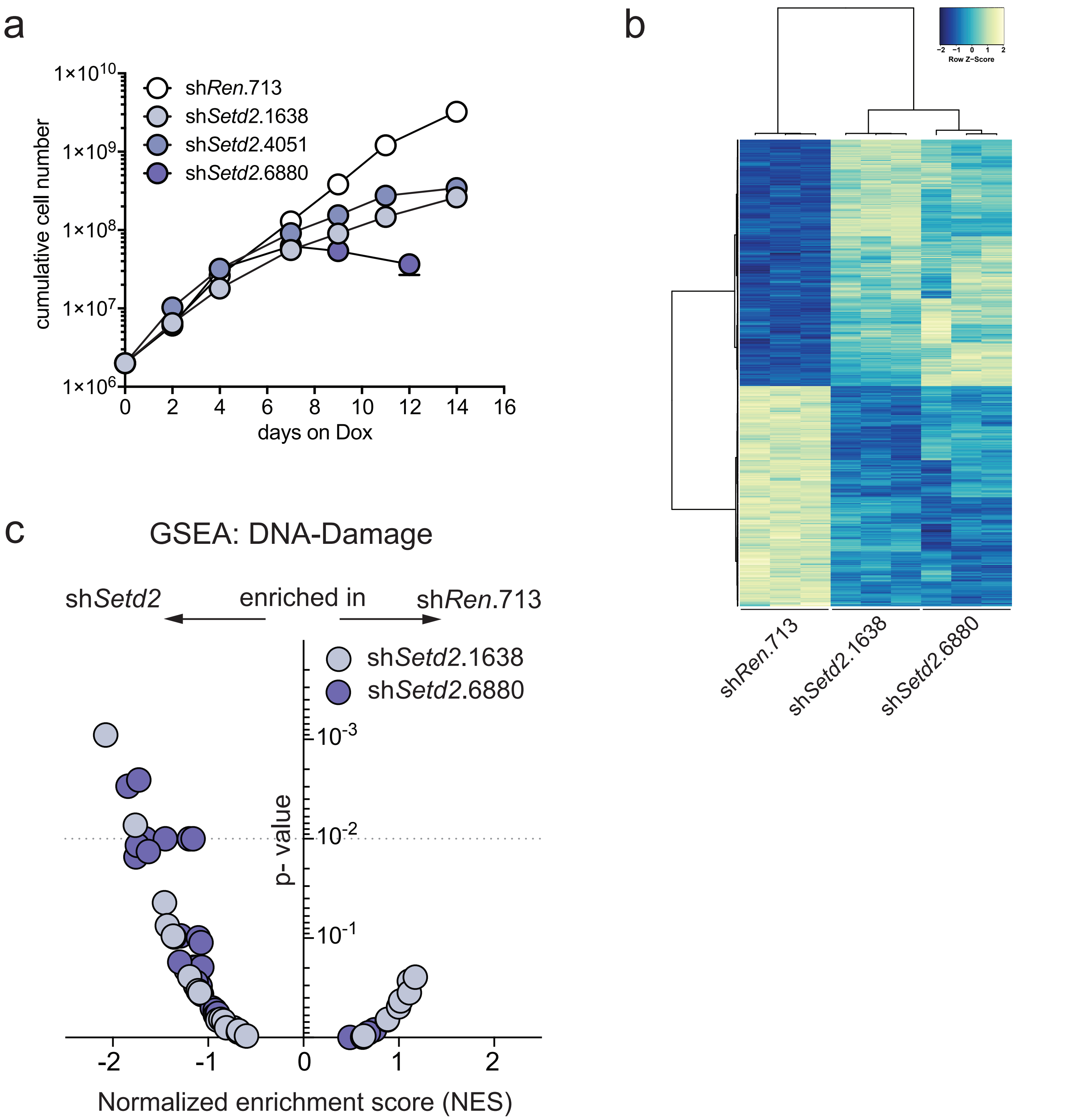


**Supplementary Figure 6. Loss of SETD2 induces DNA damage-associated transcriptional changes in MLL-AF9 expressing cells. (a)** Growth curve of mouse *MLL-AF9/NrasG12D* AML cells expressing indicated shRNAs. **(b)** Heatmap representation of differentially expressed genes in *MLL-AF9/NrasG12D* AML cells upon *Setd2* knockdown (mean±s.d. n=3). **(c)** Diagram showing results from GSEA of gene sets associated with DNA damage (from MSigDB) in *MLL-AF9/NrasG12D* AML cells upon shRNA-mediated knockdown of *Setd2*.

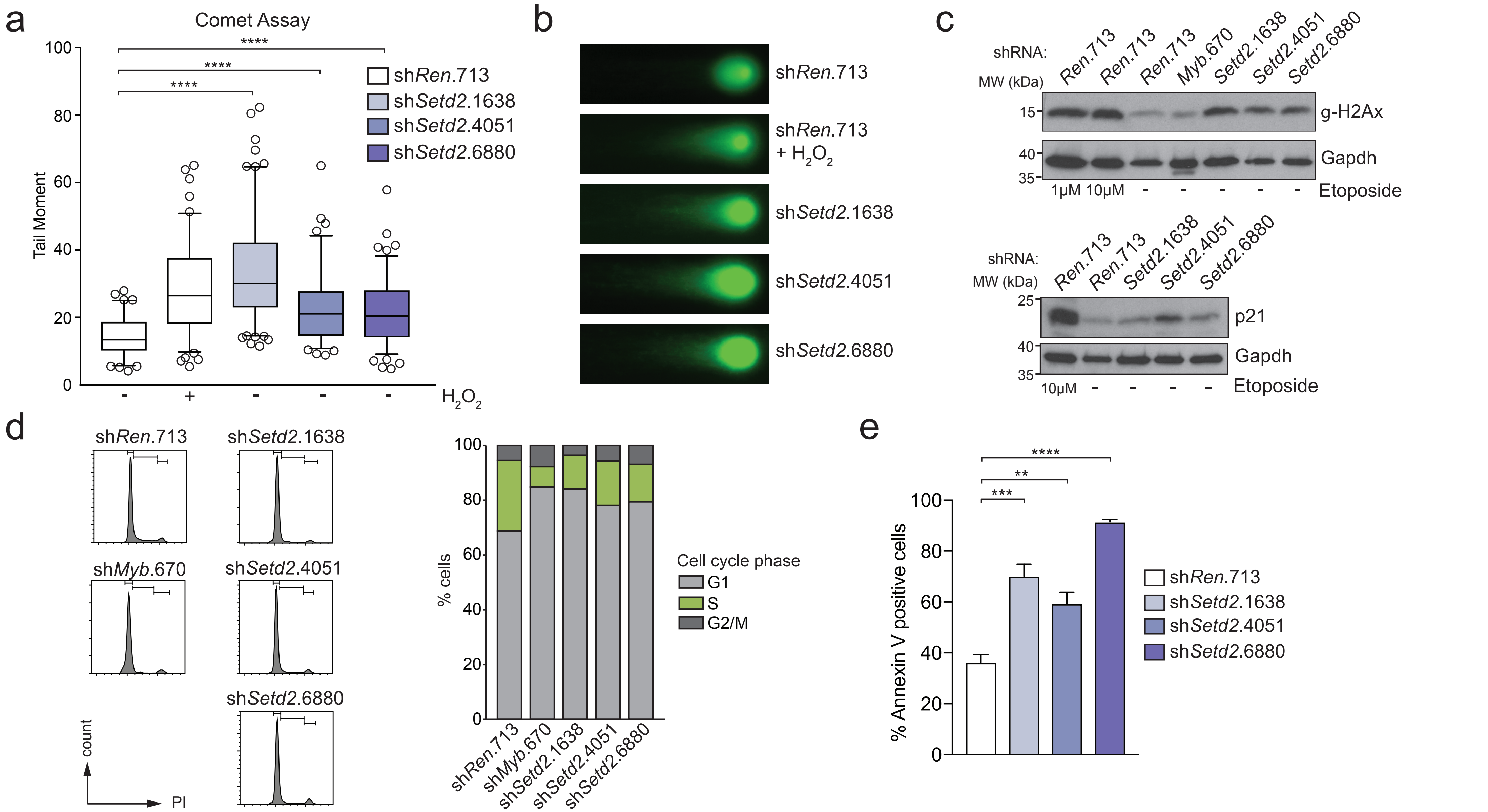


**Supplementary Figure 7. Loss of Setd2 leads to accumulation of DNA damage, p21 expression, cell cycle arrest and apoptosis in MLL-fusion-expressing cells.** **(a)** Quantification of tail moments in an alkaline comet assay performed after shRNA-mediated knockdown of *Setd2* in *MLL-AF9/NrasG12D* AML cells. Quantification of >100 cells is shown (mean±s.d.). Cells treated with 150 μM $H_2O_2$ were used as a positive control. **(b)** Representative micrographs of *MLL-AF9/NrasG12D* AML cells showing DNA breaks upon shRNA-mediated knockdown of *Setd2*. **(c)** Western blot analysis of γ-H2AX- (top) and p21 levels (bottom) in *MLL-AF9/NrasG12D* cells expressing indicated shRNAs after 3 days and 5 days of Dox treatment, respectively. Cells treated with etoposide were used a positive control. **(d)** Cell cycle analysis of *MLL-AF9/NrasG12D* AML cells expressing indicated shRNAs after Dox treatment (8 days). Histograms (left) and quantification (right) are shown. **(e)** Quantification of flow cytometric analysis of apoptosis as measured by Annexin V staining of *MLL-AF9/NrasG12D* cells expressing indicated shRNAs. Cells were treated with Dox for 9 days and processed according to the manufacturer's protocol. ** p<0.01, *** p<0.001, **** p<0.0001 (t-test) (mean±s.d. n=3).

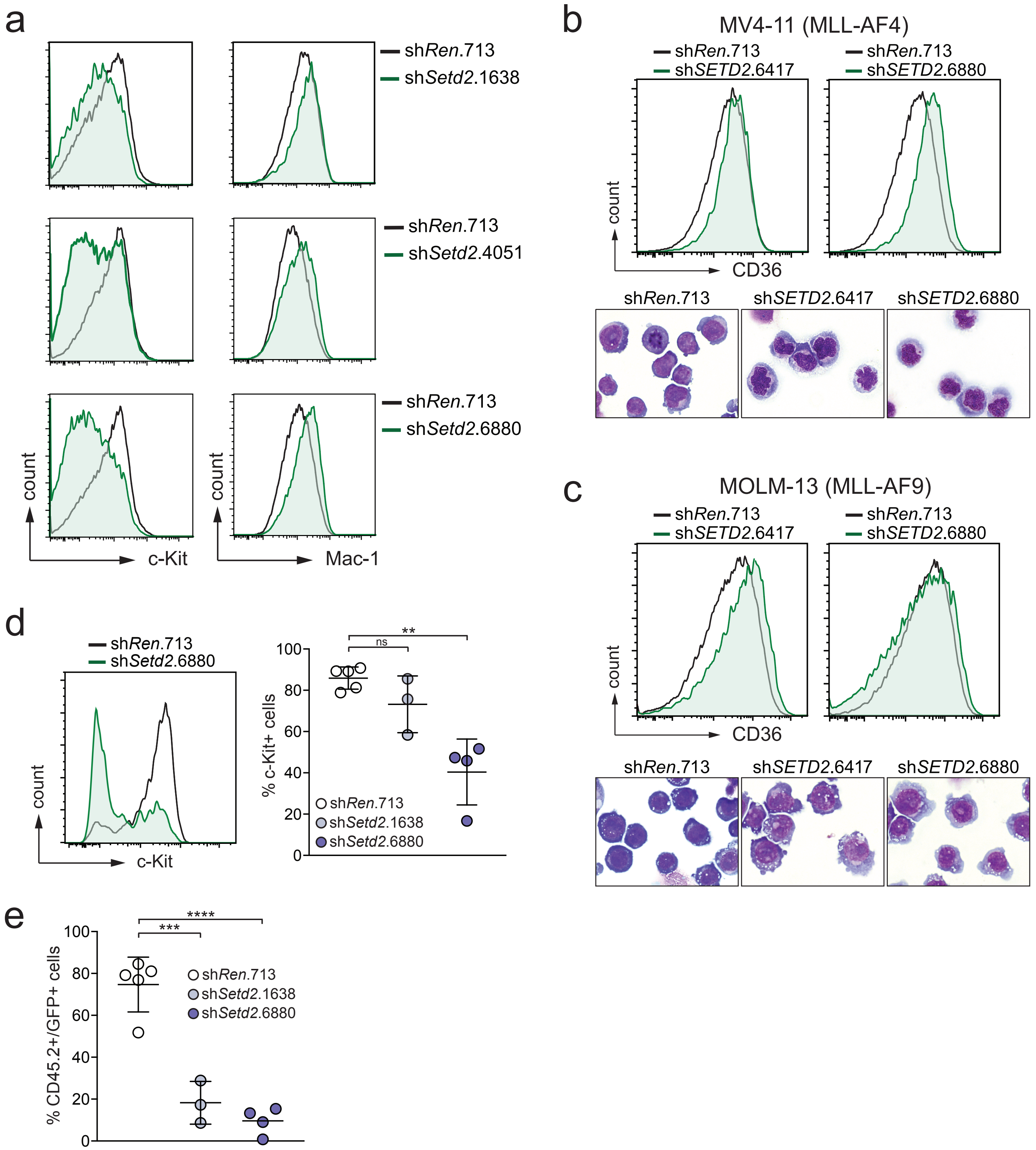


**Supplementary Figure 8. shRNA-mediated SETD2 downregulation leads to myeloid differentiation of leukemia cells *in vitro* and *in vivo*. (a)** Flow cytometric analysis of c-Kit and Mac-1 in *MLL-AF9/NrasG12D* AML cells upon *Setd2* knockdown. Data from one out of three representative experiments are shown. **(b)** Flow cytometric analysis of CD36 in MV4-11 cells upon *SETD2* knockdown (top). Data from one out of three representative experiments are shown. Micrographs of cytospin preparations of MV4-11 cells after expression of indicated shRNAs (bottom). **(c)** Flow cytometric analysis of CD36 on MOLM-13 cells upon *SETD2* knockdown (top). Data from one out of three representative experiments are shown. Micrographs of cytospin preparations of MV4-11 cells after expression of indicated shRNAs (bottom). **(d)** Flow cytometric analysis of c-Kit on *MLL-AF9/NrasG12D* AML cells upon *Setd2* knockdown *in vivo* (left). Quantification of flow cytometric analysis of c-Kit on *MLL-AF9/NrasG12D* AML cells upon *Setd2* knockdown with indicated shRNAs *in vivo* (right) (mean±s.d. n≥3) **(e)** Quantification of flow cytometric analysis of CD45.2/GFP on *MLL-AF9/NrasG12D* AML cells upon *Setd2* knockdown with indicated shRNAs *in vivo.* ns, not significant, ** $p<0.01$, *** $p<0.001$, **** $p<0.0001$ (t-test) (mean±s.d. n≥3).

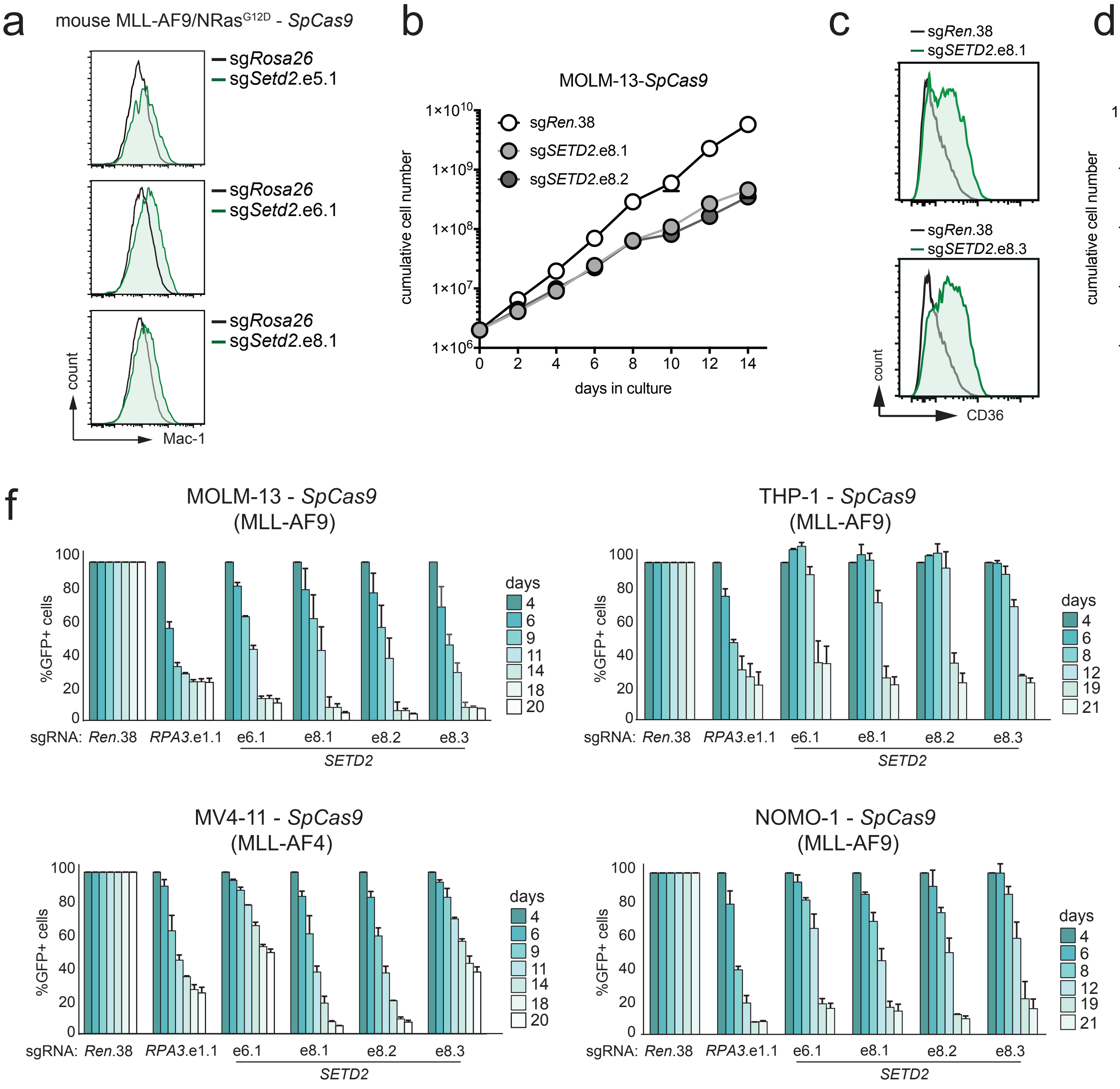


**Supplementary Figure 9. CRISPR/Cas9 -mediated mutagenesis of the SET domain of SETD2 impairs proliferation and induces differentiation of human MLL-AML cells. (a)** Flow cytometric analysis of Mac-1 in *MLL-AF9/NrasG12D-SpCas9* AML cells upon CRISPR/Cas9-mediated mutagenesis of *Setd2* with indicated sgRNAs. Data from one out of three representative experiments is shown. **(b)** Growth curve of MOLM-13-*SpCas9* cells after CRISPR/Cas9-mediated mutagenesis of *SETD2* with indicated sgRNAs (mean±s.d. n=3). **(c)** Flow cytometric analysis of surface expression of CD36 on MOLM-13-*SpCas9* cells upon CRISPR/Cas9-mediated mutagenesis of *SETD2* **(d)** Growth curve of MV4-11-*SpCas9* cells upon CRISPR/Cas9-mediated mutagenesis of *SETD2* with indicated sgRNAs (mean±s.d. n=3). **(e)** Flow cytometric analysis of surface expression of CD36 in MV4-11-*SpCas9* cells upon CRISPR/Cas9-mediated mutagenesis of *SETD2*. One representative experiment of two is shown. **(f)** Results of FACS-based competitive proliferation assay shown as the percentage of GFP-positive cells expressing individual *SETD2*-targeting sgRNAs in the presence of Dox over 20-21 days (mean±s.d. n=2).

SKUCHA_Supplementary Figure 9

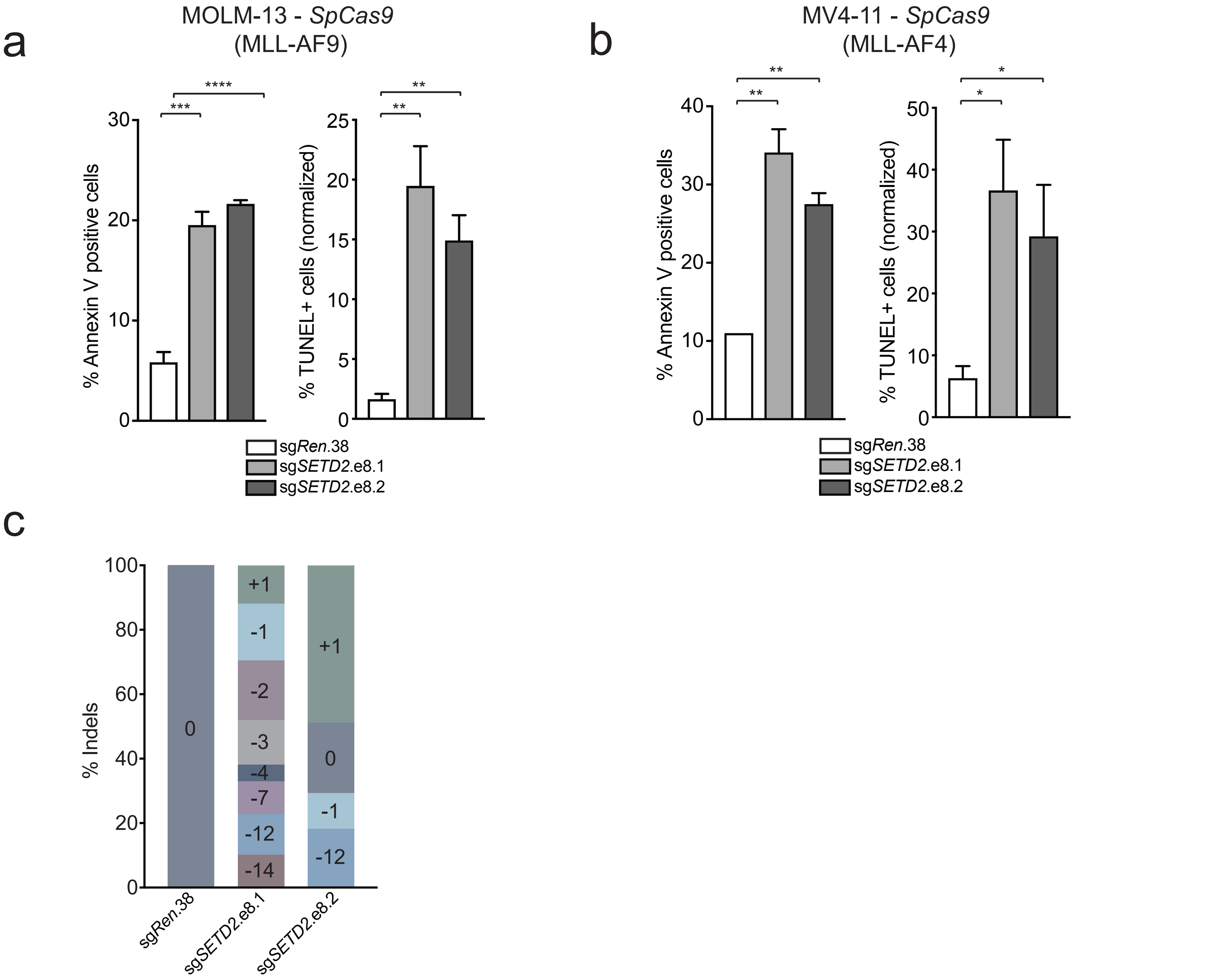


**Supplementary Figure 10. Mutagenesis of the SETD2 SET domain leads to apoptosis of MLL-fusion expressing cells. (a)** Flow cytometric apoptosis detection as measured by Annexin V- (left) and TUNEL staining (right) in MOLM-13-*SpCas9* cells upon CRISPR/Cas9-mediated mutagenesis of *SETD2* with indicated sgRNAs (mean±s.d. n=3). **(b)** Flow cytometric apoptosis detection as measured by Annexin V- (left) and TUNEL staining (right) in MV4-11-*SpCas9* cells upon CRISPR/Cas9-mediated mutagenesis of *SETD2* with indicated sgRNAs (mean±s.d. n=3). **(c)** Analysis of indel formation by *SETD2*-targeting sgRNAs in MV4-11-*SpCas9* cells using Sanger sequencing and TIDE analysis. * p>0.05, ** p<0.01, *** p<0.001, **** p<0.0001 (t-test).

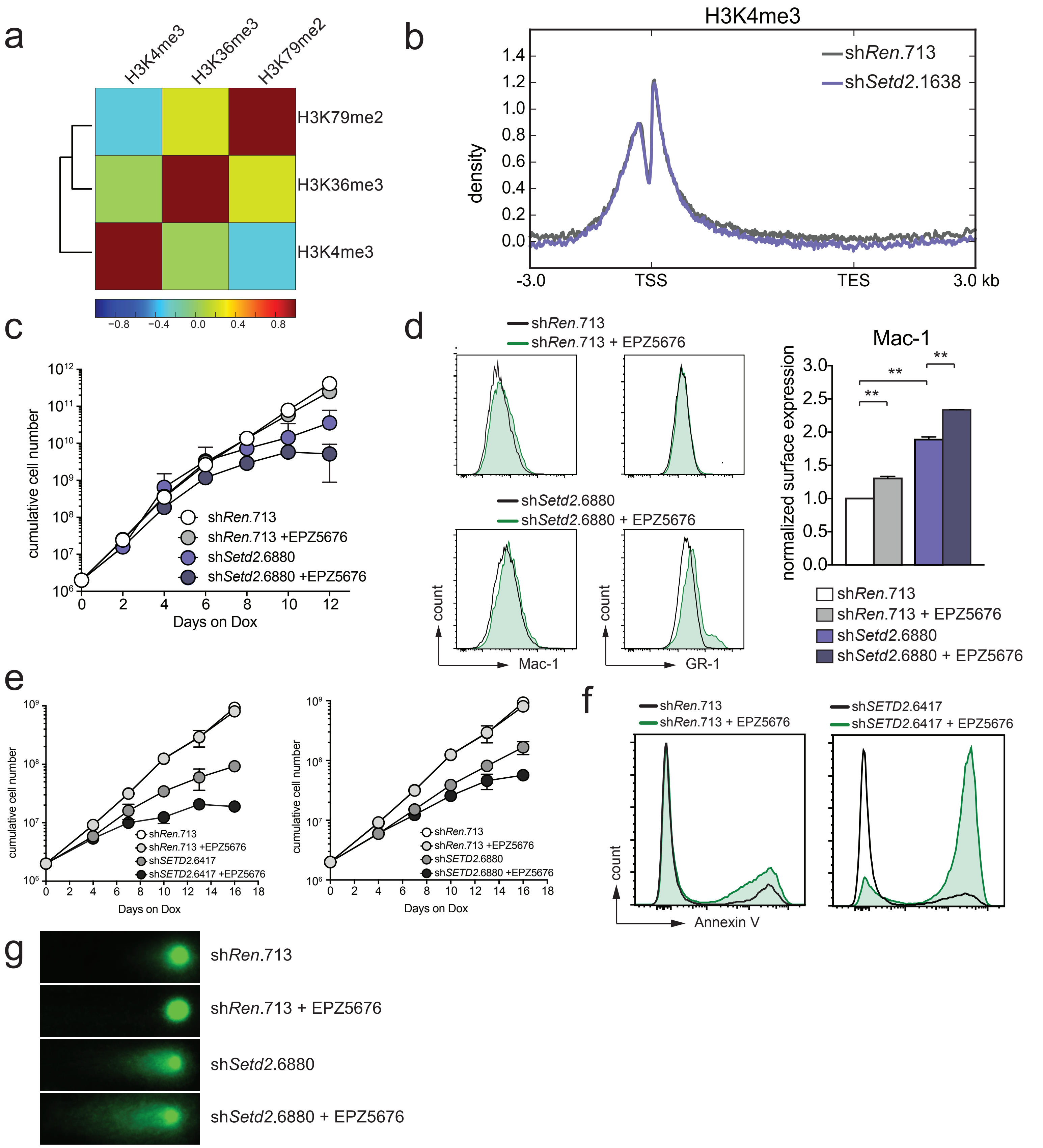


**Supplementary Figure 11. SETD2 loss disrupts the H3K36me3-H3K79me2 signature on MLL-target genes and sensitizes AML cells to DOT1L inhibition. (a)** Heatmap representation of the Pearson correlation coefficients between changes in the respective histone marks over gene bodies from ChIP-Rx experiments after shRNA-mediated knockdown of *Setd2* in *MLL-AF9/NrasG12D* cells. **(b)** Metagene plots of ChIP-Rx data for H3K4me3 after *Setd2* knockdown. **(c)** Growth curves of *MLL-AF9/NrasG12D* AML cells treated with EPZ5676 (500 nM) upon shRNA-mediated mutagenesis of *Setd2* with indicated shRNAs (mean±s.d. n=3). **(d)** Flow cytometric analysis of Mac-1 and Gr-1 expression in *MLL-AF9/NrasG12D* cells treated with EPZ5676 (500nM) upon shRNA-mediated knockdown of *Setd2* (left). Quantification of surface expression of Mac-1 on *MLL-AF9/NrasG12D* AML cells treated with EPZ5676 (500nM) after shRNA-mediated knockdown of *Setd2* (mean±s.d. n=2) (right). **(e)** Growth curves of MV4-11 cells upon shRNA-mediated knockdown of *SETD2* with indicated shRNAs treated with EPZ5676 (50nM) (mean±s.d. n=3). **(f)** Flow cytometric analysis of apoptosis induction as measured by Annexin V-staining in MV4-11 cells treated with EPZ5676 (50nM) after shRNA-mediated knockdown of *SETD2*. **(g)** Representative micrographs of *MLL-AF9/NrasG12D* AML cells showing DNA breaks after treated with EPZ5676 (500nM) upon shRNA-mediated knockdown of *Setd2*. ** $p<0.01$ (t-test).

**Supplementary Figure 12.** Full, uncropped scans of Western blots

Figure 1a

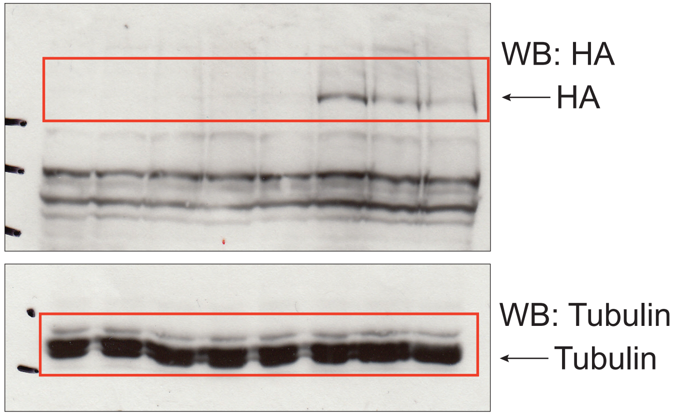


Supplementary Figure 1a

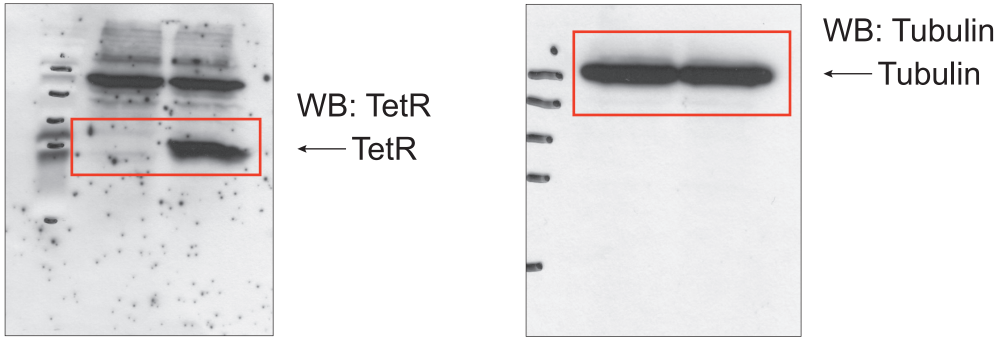


Supplemenary Figure 1d

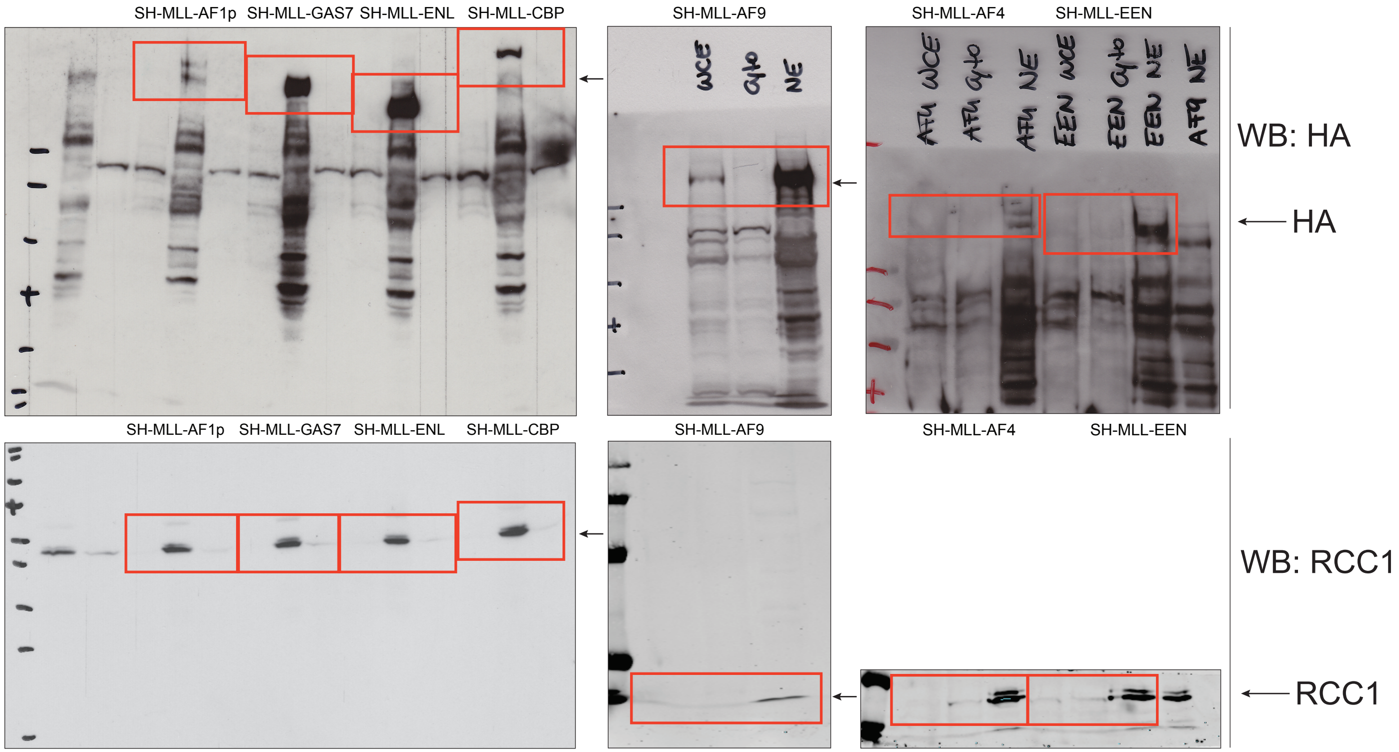


Supplementary Figure 2

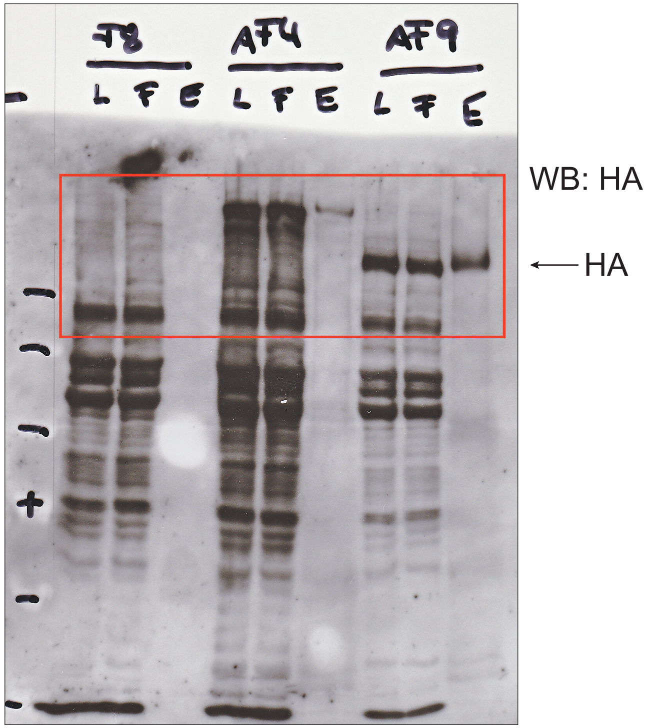

Supplemenary Figure 3c

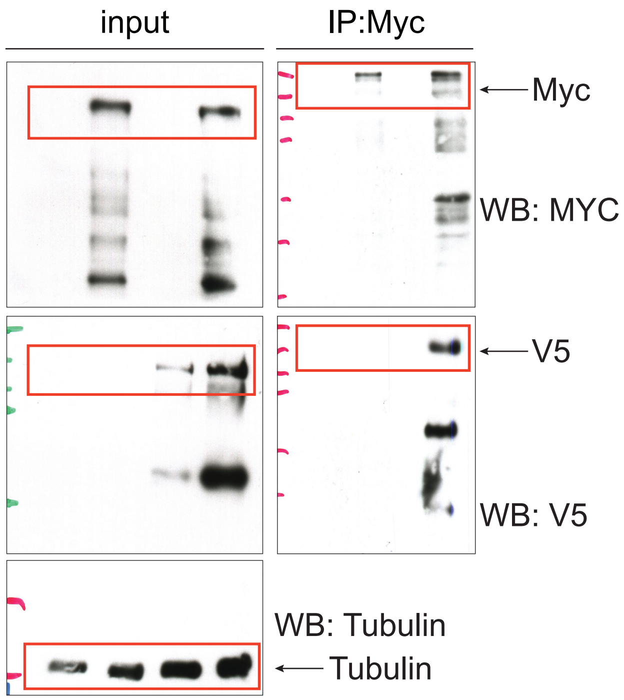


Figure 3c

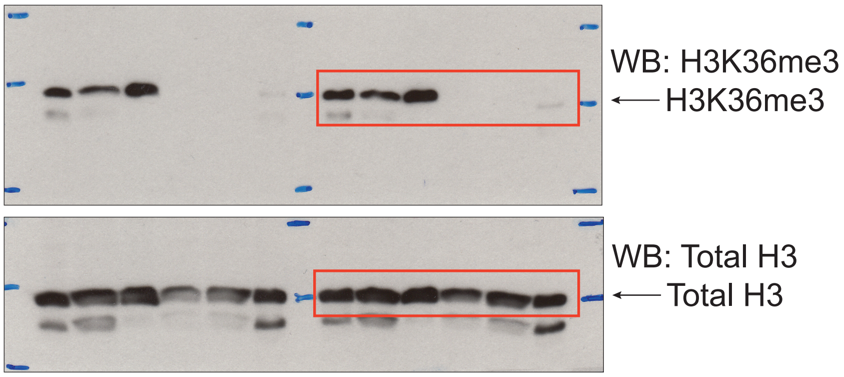


Figure 5c

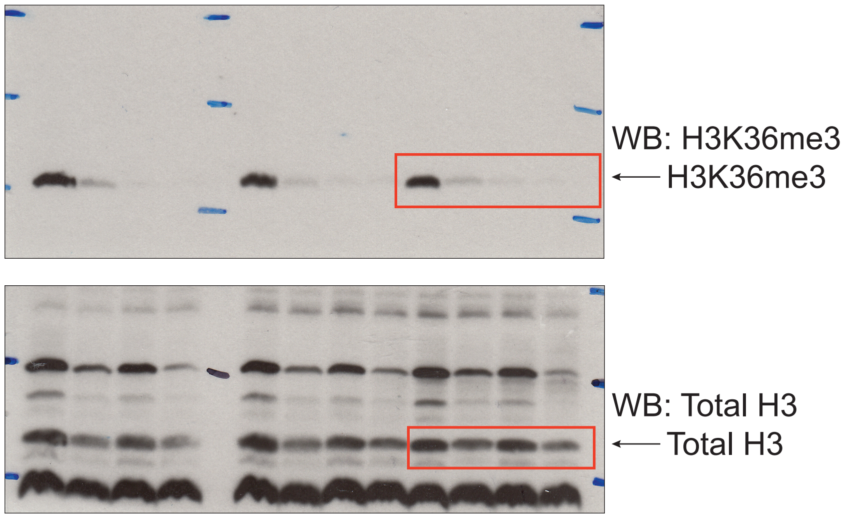


Supplementary Figure 7c

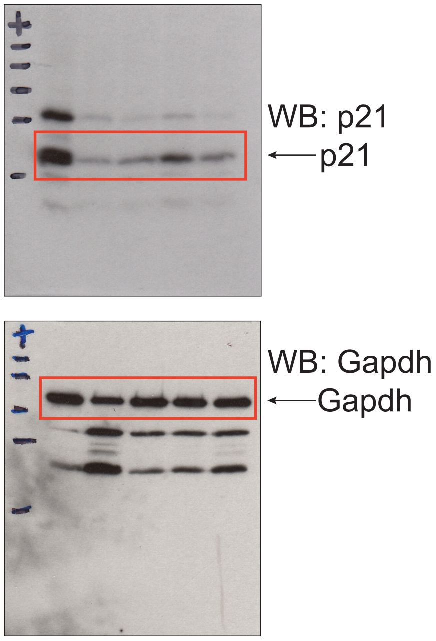


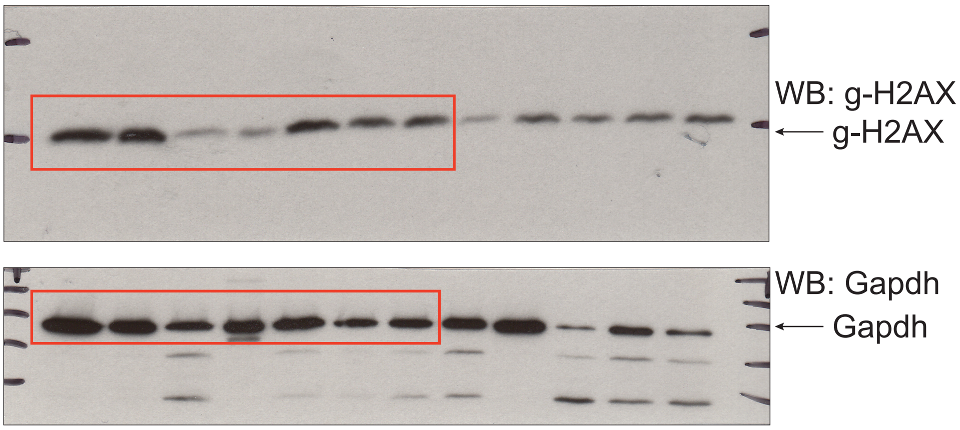

**Supplementary Table 1**. Functional annotation of conserved MLL-fusion interactors

| Community:1<br>GO.id | <br>Term | <br>Pvalue | <br>Adj.Pvalue |
|---|---|---|---|
| GO:0051084 | 'de novo' posttranslational protein folding | 2.40E-09 | 1.80E-07 |
| GO:0043044 | ATP-dependent chromatin remodeling | 1.00E-08 | 3.60E-07 |
| GO:0045944 | positive regulation of transcription from RNA pol II promoter | 1.60E-05 | 0.00023 |
| GO:0060216 | definitive hemopoiesis | 4.00E-04 | 0.00392 |
| GO:0035162 | embryonic hemopoiesis | 0.00043 | 0.00392 |
| GO:0044648 | histone H3-K4 dimethylation | 0.00124 | 0.00766 |
| Community:2 | purple | | |
| GO.id | Term | Pvalue | Adj.Pvalue |
| GO:0016925 | protein sumoylation | 6.80E-09 | 5.20E-07 |
| GO:0019054 | modulation by virus of host process | 3.30E-08 | 1.30E-06 |
| GO:0007077 | mitotic nuclear envelope disassembly | 3.10E-06 | 7.00E-05 |
| GO:0043687 | post-translational protein modification | 4.20E-06 | 7.00E-05 |
| GO:0010827 | regulation of glucose transport | 3.80E-05 | 0.00041 |
| GO:0031047 | gene silencing by RNA | 7.20E-05 | 0.00061 |
| Community:3 | green | | |
| GO.id | Term | Pvalue | Adj.Pvalue |
| GO:0006977 | DNA damage response, signal transduction by p53 | 3.70E-07 | 3.50E-05 |
| GO:0000278 | mitotic cell cycle | 8.40E-06 | 0.00028 |
| GO:0007411 | axon guidance | 8.90E-06 | 0.00028 |
| GO:0031145 | anaphase-promoting complex-dependent process | 1.50E-05 | 0.00036 |
| GO:0006521 | regulation of cellular amino acid metabolic process | 2.70E-05 | 0.00051 |
| GO:0051437 | positive regulation of ubiquitin-protein ligase activity involved in regulation of mitotic cell cycle transition | 4.00E-05 | 0.00063 |
| Community:4 | orange | | |
| GO.id | Term | Pvalue | Adj.Pvalue |
| GO:0000184 | nuclear-transcribed mRNA catabolic process | 6.00E-09 | 3.20E-07 |
| GO:0006413 | translational initiation | 3.00E-05 | 0.00041 |
| GO:0006364 | rRNA processing | 3.40E-05 | 0.00041 |
| GO:0006415 | translational termination | 6.20E-05 | 0.00053 |
| GO:0000086 | G2/M transition of mitotic cell cycle | 7.70E-05 | 0.00053 |
| GO:0006414 | translational elongation | 0.00012 | 0.00072 |
| Community:5 | red | | |
| GO.id | Term | Pvalue | Adj.Pvalue |
| GO:0000398 | mRNA splicing, via spliceosome | 7.70E-13 | 1.30E-11 |
| GO:0008380 | RNA splicing | 1.70E-06 | 1.40E-05 |
| GO:0006397 | mRNA processing | 4.20E-06 | 2.40E-05 |
| GO:0006369 | termination of RNA polymerase II transcription | 1.30E-05 | 5.50E-05 |
| GO:0006378 | mRNA polyadenylation | 0.00069 | 0.00235 |
| GO:0000349 | generation of catalytic spliceosome for first transesterification step | 0.00101 | 0.00286 |
| Community:6 | ligth blue | | |
| GO.id | Term | Pvalue | Adj.Pvalue |

| | | | |
|---|---|---|---|
| GO:0043983 | histone H4-K12 acetylation | 3.90E-09 | 5.90E-08 |
| GO:0043981 | histone H4-K5 acetylation | 3.20E-08 | 1.60E-07 |
| GO:0043982 | histone H4-K8 acetylation | 3.20E-08 | 1.60E-07 |
| GO:0043984 | histone H4-K16 acetylation | 1.80E-07 | 6.70E-07 |
| GO:0043966 | histone H3 acetylation | 2.70E-06 | 8.10E-06 |
| GO:0006270 | DNA replication initiation | 0.00011 | 0.00028 |

**Supplementary Table 2.** sgRNA sequences targeting SETD2 and controls used in this study

| target | sequence |
|---|---|
| Ren.38 | GGATGATAACTGGTCCGCAGTGG |
| RPA3 e1.1 | CCGGCGTTGATGCGCGACCTGGG |
| SETD2.e6.1 | AGAGTTTAAAGCTCGAGTGAAGG |
| SETD2.e8.1 | GGACTGTGAACGGACAACTGAGG |
| SETD2.e8.2 | GACTGTGAACGGACAACTGAGGG |
| SETD2.e8.3 | GTGAACGGACAACTGAGGGTTGG |

| target | sequence |
|---|---|
| Rosa26 | GAAGATGGGCGGGAGTCTTC |
| Myb.33 | GCTGAAGAAGCTGGTGGAACAGAACGGAAC |
| Setd2_e1.1 | AAATCCCCCATCTTCGGTGG |
| Setd2_e3.1 | TCAAGTCGATTTTTGCCCAA |
| Setd2_e5.1 | CTGGGGCTTAAGGGCTGCTA |
| Setd2_e6.1 | GTACCTCTCCACAGTATTCC |
| Setd2_e8.1 | GCCTGAAGGAACTAGTTTGG |

**Supplementary Table 3.** shRNA sequences targeting SETD2 and controls used in this study

| target | sequence |
|---|---|
| Ren.713 | TGCTGTTGACAGTGAGCGCAGGAATTATAATGCTTATCTATAGTGAAGCCACAGATGTATAG ATAAGCATTATAATTCCTATGCCTACTGCCTCGGA |
| MEN1.1105 | TGCTGTTGACAGTGAGCGATACAAGGAGTTCTTTGAAGTATAGTGAAGCCACAGATGTATAC TTCAAAGAACTCCTTGTAGTGCCTACTGCCTCGGA |
| MLL.3710 | TGCTGTTGACAGTGAGCGCAGAGAGCAGTGTTGTGAAGAATAGTGAAGCCACAGATGTATT CTTCACAACACTGCTCTCTTTGCCTACTGCCTCGGA |
| MYB.721 | CTCGAGAAGGTATATTGCTGTTGACAGTGAGCGACTGGACGAACTGATAATGCTATAGTGAA GCCACAGATGTATAGCATTATCAGTTCGTCCAGGTGCCTACTGCCTCGGAATTC |
| SETD2.124 | TGCTGTTGACAGTGAGCGCGAAGAAGAAGAAAATGAGGCATAGTGAAGCCACAGATGTATG CCTCATTTTCTTCTTCTTCATGCCTACTGCCTCGGA |
| SETD2.4318 | TGCTGTTGACAGTGAGCGCCAGGACAGAAAGAAAGTTAGATAGTGAAGCCACAGATGTATC TAACTTTCTTTCTGTCCTGATGCCTACTGCCTCGGA |
| SETD2.4834 | TGCTGTTGACAGTGAGCGAAAGGAGTATGCACGAAACAAATAGTGAAGCCACAGATGTATTT GTTTCGTGCATACTCCTTCTGCCTACTGCCTCGGA |
| SETD2.6417 | TGCTGTTGACAGTGAGCGACCGGAAGTTGTTTGAGCAAGATAGTGAAGCCACAGATGTATCT TGCTCAAACAACTTCCGGCTGCCTACTGCCTCGGA |
| SETD2.6880 | TGCTGTTGACAGTGAGCGCAACCAACAGTCTGTCAGTGTATAGTGAAGCCACAGATGTATAC ACTGACAGACTGTTGGTTTTGCCTACTGCCTCGGA |
| SETD2.7461 | TGCTGTTGACAGTGAGCGCAAGCAAAGAAGTATTCAGAAATAGTGAAGCCACAGATGTATTT CTGAATACTTCTTTGCTTTTGCCTACTGCCTCGGA |

| | |
|---|---|
| Myb.670 | CTCGAGAAGGTATATTGCTGTTGACAGTGAGCGACACAACCATTTGAATCCAGAATAGTGAA GC CACAGATGTATTCTGGATTCAAATGGTTGTGCTGCCTACTGCCTCGGAATTC |
| Setd2.1638 | TGCTGTTGACAGTGAGCGCAACGGACTTCAGAGCATGAAATAGTGAAGCCACAGATGTATTT CATGCTCTGAAGTCCGTTTTGCCTACTGCCTCGGA |
| Setd2.4051 | TGCTGTTGACAGTGAGCGACAGACTGAAGATTATAAGTGATAGTGAAGCCACAGATGTATCA CTTATAATCTTCAGTCTGCTGCCTACTGCCTCGGA |
| Setd2.6880 | TGCTGTTGACAGTGAGCGCAACCAACAGTCTGTCAGTGTATAGTGAAGCCACAGATGTATAC ACTGACAGACTGTTGGTTTTGCCTACTGCCTCGGA |

**Supplementary Table 4**. Primers used in this study

| **qPCR primers used in this study** | | |
|---|---|---|
| mSetd2_1 | Forward | GATCAAACAGCTCCAAAGACTC |
| | Reverse | GGTGTATCATATCTGTCATCTGG |
| mSetd2_2 | Forward | CTTATTCACAGACAACTCCACC |
| | Reverse | CATTTCAGATGGCTGCAAGG |
| mHoxA9 | Forward | GCAGTGTATCATCACCACCA |
| | Reverse | TTTCTCTATCAACTGCCGTCC |
| hSETD2 | Forward | AAACACAACAACTGAACGAGG |
| | Reverse | GAGTTTGCTTGTCTGGGTCTC |

| **ChIP qPCR primers used in this study** | | |
|---|---|---|
| mHoxA9 | Forward | GCACTGGACTTGAGCTGTAGTTTG |
| | Reverse | CCCCTGCCTTGGTTATCCTT |
| mMeis1 | Forward | CAGGCGGAGAGGTGGATTC |
| | Reverse | CCTGCCGAGGAGCCTACTTG |

| **Primers used for genotyping of cells with CRISPR/Cas9-induced mutations in SETD2** | |
|---|---|
| forward | GCACACACACACACACACAC |
| reverse | CTCCTCAACTTCATATGGTCTACT |
