# Supplementary material for: MLL-fusion-driven leukemia requires SETD2 to safeguard genomic integrity
Source: Nat Commun. 2018 May 18;9:1983. doi: 10.1038/s41467-018-04329-y (PMC5959866; doi:10.1038/s41467-018-04329-y)
Supplement: Supplementary file 3 — Description of Additional Supplementary Files [file 41467_2018_4329_MOESM3_ESM.pdf]

Description of Additional Supplementary Files

File Name: Supplementary Data 1
Description: Spectral counts values of 960 interactors of 7 selected MLL fusion proteins

File Name: Supplementary Data 2
Description: shRNA sequences targeting 128 conserved interactors of seven
